# Outward Rectification of Voltage-Gated K^+^ Channels Evolved at Least Twice in Life History

**DOI:** 10.1371/journal.pone.0137600

**Published:** 2015-09-10

**Authors:** Janin Riedelsberger, Ingo Dreyer, Wendy Gonzalez

**Affiliations:** 1 Centro de Bioinformática y Simulación Molecular (CBSM), Universidad de Talca, Talca, Chile; 2 Centro de Biotecnología y Genómica de Plantas, Universidad Politécnica de Madrid, Madrid, Spain; 3 Institute for Biochemistry and Biology, University of Potsdam, Potsdam, Germany; Zhejiang University, CHINA

## Abstract

Voltage-gated potassium (K^+^) channels are present in all living systems. Despite high structural similarities in the transmembrane domains (TMD), this K^+^ channel type segregates into at least two main functional categories—hyperpolarization-activated, inward-rectifying (K_in_) and depolarization-activated, outward-rectifying (K_out_) channels. Voltage-gated K^+^ channels sense the membrane voltage via a voltage-sensing domain that is connected to the conduction pathway of the channel. It has been shown that the voltage-sensing mechanism is the same in K_in_ and K_out_ channels, but its performance results in opposite pore conformations. It is not known how the different coupling of voltage-sensor and pore is implemented. Here, we studied sequence and structural data of voltage-gated K^+^ channels from animals and plants with emphasis on the property of opposite rectification. We identified structural hotspots that alone allow already the distinction between K_in_ and K_out_ channels. Among them is a loop between TMD S5 and the pore that is very short in animal K_out_, longer in plant and animal K_in_ and the longest in plant K_out_ channels. In combination with further structural and phylogenetic analyses this finding suggests that outward-rectification evolved twice and independently in the animal and plant kingdom.

## Introduction

**Fig 1:**
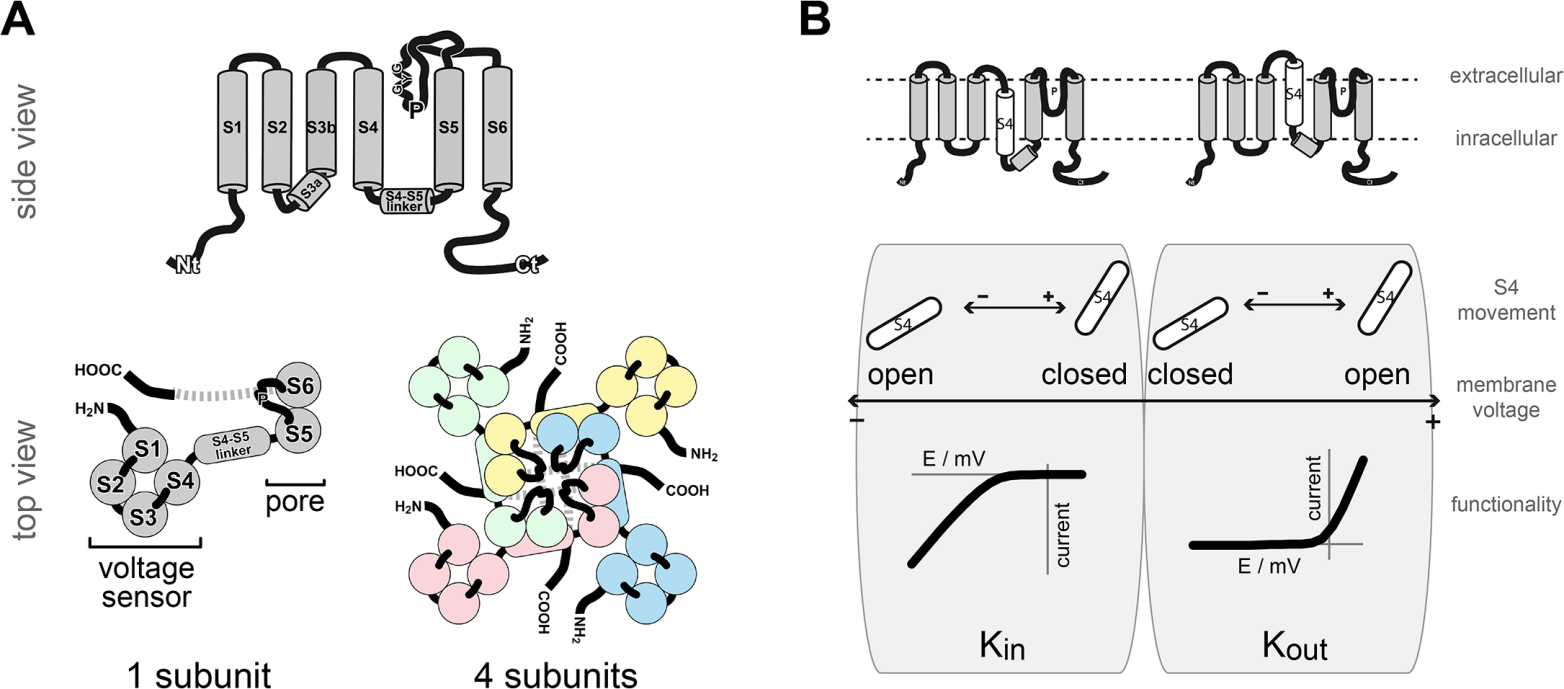
Structure and function of voltage-gated K^+^ channels (A) Functional voltage-gated K^+^ channels form tetramers (coloured representation). Each subunit (grey representation) comprises six transmembrane domains (S1-S6) and a selectivity filter between S5 and S6. The first four TMDs form the voltage sensor that is located in the periphery of the functional channel. The last two TMDs contribute to the channel pore in the centre of the channel. (B) TMD S4 of the voltage sensor translocates in response to the transmembrane voltage within the membrane and affects the channel conformation. It moves towards the intracellular side of the membrane upon hyperpolarizing membrane voltages and towards the extracellular side upon membrane depolarization. The “down” movement of S4 causes K_in_ channels to open, while the same S4 movement results in closure of K_out_ channels. The opening of K_out_ and closure of K_in_ channels proceeds in the inverse manner in response to S4 “up” movements.

Voltage-gated potassium (K^+^) channels have been investigated in deep detail in various organisms ranging from pro- to eukaryotic species (reviewed in [1–4]). Having a common subunit structure, these channels consist of six transmembrane domains (TMD), S1 to S6, and a pore helix and selectivity filter between the last two TMD (S5–P–S6) (Fig 1A). Functional channels are tetramers, in which the four subunits are twisted with each other. The S5-P-S6 parts act together in the centre of the channel and form the ion conduction pathway. TMDs S1-S4 form the voltage sensors that are located in the periphery of the channel. They are connected to the pore via a short amino acid sequence, the S4-S5 linker.

Our current picture of channel gating assumes that the fourth TMD (S4) moves in a voltage-dependent manner within the membrane (Fig 1B, top). This movement is forwarded to the pore and results in conformational changes that cause the pore to open or close [5]. Initially, the S4-S5 linker was supposed to transmit the S4 movement to the pore and to couple both channel modules [6,7]. Recent evidence, however, indicates that this picture is probably too simple. Instead, the interaction of the S4-S5 linker with the C-terminal end of S6 has been described to be crucial for gating of various voltage-gated potassium channels [8–10]. Additionally, a second connection between the voltage sensor and the pore has been discovered on the extracellular channel side with particular importance for channel functionality [11]. In contrast to the intracellular interface (S4-S5 linker) that connects voltage sensor and pore on the primary protein structure level, the extracellular interface is established on the tertiary protein structure level by interacting amino acid side chains. The function of the extracellular interface is described as a fix point that is necessary to enable the intracellular interface to act as a mechanical lever on the pore’s gate. During channel gating the S4 movement shifts the mechanical lever, while the extracellular interface provides the structural stability within the channel to allow an S4 movement relative to the pore.

Voltage-gated potassium channels appear in at least two different functional types–hyperpolarization-activated, inward-rectifying (K_in_) and depolarization-activated, outward-rectifying (K_out_) K^+^ channels. For practical reasons, we use here in this study the unified nomenclature of K_in_ and K_out_ channels that is well established for voltage-gated K^+^ channels of plants [12]. We should emphasize, however, that inward-rectifying K_in_ channels must not be confused with two transmembrane domain Kir channels (also called ‘inward-rectifying K^+^ channels’), which gain their rectification properties by a voltage-dependent block and not by the movement of a voltage sensor [13,14].

In the animal kingdom, we identified two channel families that can be classified as K_in_ and K_out_ channels, respectively. The Kv family represents animal K_out_ channels and the HCN family might be considered as animal K_in_ channels. Strictly speaking, HCN channels are cation channels that possess also a significant permeability to sodium (Na^+^). Nevertheless, potassium permeates to a much larger extent, and in the absence of K^+^ HCN channels hardly transport Na^+^ [1,15]. Besides, HCN channels show the same six TMD structure, including the voltage sensor, as animal Kv and plant voltage-gated K^+^ channels, and contain the selectivity filter motif GYG that is present in highly selective potassium channels. Because HCN channels are furthermore voltage-gated and hyperpolarization activated as it has been described for plant K_in_ channels it appears to be appropriate to include this channel class in our comparative analyses. For the sake of simplicity, we refer to HCN channels as animal K_in_ channels.

In plants the family of voltage-gated K^+^ channels combines both, K_in_ and K_out_ channels, together with two other functional types: weak-rectifying (K_weak_) K^+^ channels and functionally silent regulatory subunits [3]. The first voltage-gated K^+^ channels in plants had been described to be structurally similar to animal Kv channels [16–18]. Therefore, in literature the term “plant Kv-like channels” has widely been established when referring to the group of plant voltage-gated K^+^ channels. So we will use this term in the following as well.

The response of voltage-gated K^+^ channels to a change in the transmembrane voltage begins with the transition of the fourth TMD. Interestingly, S4’s movement is conserved in these K^+^ channels. It has been shown experimentally that S4 moves downward upon hyperpolarizing voltages and upward upon depolarizing membrane voltages [19]. Curiously, the same S4 movement results in opening of K_in_ channels, while it results in the closure of K_out_ channels and vice versa (Fig 1B, bottom) [20,21]. Because the fundamental voltage sensing mechanism is not different, it is hypothesized that the coupling of voltage sensor and pore must be different in both channel types. Astonishingly, the main structural components that are important for channel gating, like voltage sensor, S4-S5 linker and pore, are quite conserved in voltage-gated K^+^ channels and bear only slight differences [12]. Thus, coupling of voltage sensor and pore as well as the opposite rectification of K_in_ and K_out_ channels seem to be subtly implemented within the channel structure.

In this study we analysed and compared sequence and structural data of voltage-gated K^+^ channels with a focus on the four groups of animal K_in_ (HCN), animal K_out_ (Kv), plant K_in_ and plant K_out_ (plant Kv-like) channels. The results allowed us (i) to reason an independent evolution of outward-rectification in plants and animals and (ii) to develop a more sophisticated picture on voltage-gated K^+^ channel gating that might be suited to account for differences in gating of these channels.

## Material and Methods

### Sequence data analyses

Known sequences of voltage-gated K^+^ channels were extracted from databases indicated in tables and figures. Putative sequences of K^+^ channels from *Klebsormidium flaccidum* were extracted from http://www.plantmorphogenesis.bio.titech.ac.jp/~algae_genome_project/klebsormidium/index.html [22]. Sequences of the plant K_in_ channel KAT1 and the plant K_out_ channel SKOR were blasted against the database. Those sequences have been selected that contained features characteristic for voltage-gated K^+^ channels, like the selectivity filter motif GYGD, positive charges in S4 and the S4-S5 linker as well as characteristic residues in the C-terminal end of S6.

The multiple sequence alignment (MSA) of K^+^ channel sequences was performed using ProbCons [23]. Subsequent phylogenetic analysis was carried out via MEGA6 by using the Maximum Likelihood method based on the amino acid replacement model developed by Whelan And Goldman (WAG). The WAG model combines attributes of the amino acid replacement models of Dayhoff and JTT and has been shown to provide a better overall fit to the evolutionary process [24,25]. Initial trees for the heuristic search were obtained by applying the Neighbour-Joining method to a matrix of pairwise distances estimated using a JTT model. A resampling analysis with 1000 bootstrap replications was applied and all positions with less than 15% site coverage were eliminated during tree construction.

Clustering analyses were performed by calculating the average distance between sequences using the substitution matrix BLOSUM62. Analyses were executed through the function ‘Average Distance Using BLOSUM62’ that is implemented in JalView on the basis of the previous obtained MSA [26]. Resulting trees were loaded in MEGA6 to prepare final tree representation. For average distance analyses on sequence fragments the full-length sequences were separated according to the proposed position of transmembrane domains provided by the multiple sequence alignment. New MSA were executed for fragmented sequences and average distance trees calculated as described for full-length sequences.

To calculate the conservation of sequence parts the conservation values given by JalView were used. For each column in the multiple sequence alignment a value between 0 (not conserved) and 11 (100% conserved) is assigned. The average of these values was calculated for the columns representing the N- and C-termini and the core channel structure, respectively. Average conservation of the cytosolic termini was 0.05, while the average conservation of the core channel part was 1.82. Thus, the core region shows a more than 30 times higher conservation than the termini.

### Structural data analyses

The protein crystal structure of Kv1.2 in the open state (2A79) was downloaded from www.rcsb.org. Kv1.2 in the closed state [27] as well as KAT1 and SKOR in both conformational states [28] are only available as modelled protein structures. For the KAT1 and SKOR models 6ns of molecular dynamics simulation are available.

Analyses of protein structures were performed in VMD by executing tcl-scripts to calculate the data described in the following. Potential interactions between voltage sensor and pore on the extra- and intracellular side were identified by searching for residues that are within 3Å of residues in the C-terminal end of S1 or the S4-S5 linker, respectively. The diameter of the ring that is formed by the four S4-S5 linkers was calculated by measuring the distance of two opposite residues from the middle of the linker using the function ‘bond’ from VMD. With four linkers this calculation resulted in two distances, whose mean was calculated to determine the final S4-S5 linker ring diameter. The ‘bond’ function was also used to calculate the distance of neighbouring S6 ends. The mean was calculated of four distances to determine the average distance of S6 ends. To calculate the bend of S6 three points were defined within S6: One point in the bend around the PxP motive (animal Kv) or the conserved glycine (HCN, plant Kv-like), and two more at the beginning and the end of S6. To place the points in the centre of the α-helix three amino acids were selected and the centre between them measured. Subsequently, the angle at the point in the bend was measured by calculating the angle between the two lines that arise when the middle point is connected with the points at both ends of S6.

## Results and Discussion

For our comparison of voltage-gated potassium (K^+^) channels from plants and animals we used as prototypes the nine Kv-like channels from the model plant *Arabidopsis thaliana*, 12 hyperpolarization-activated cyclic nucleotide-gated cation channels (HCNs) and 10 Kv channels from *Rattus norvegicus*, *Mus musculus* and *Homo sapiens*, as well as the HCN from the sea urchin, *Strongylocentrotus purpuratus*, and the Shaker channel from *Drosophila melanogaster*. According to their voltage-dependence, these channels were categorized into hyperpolarization-activated, inward-rectifying (K_in_) and depolarization-activated, outward-rectifying (K_out_) channels [12]. Based on their voltage-dependence and on their highest permeability for K^+^, HCN channels are considered as animal K_in_ channels although they are no strict K^+^ channels but also permeable to other cations, like e.g. Na^+^ [15]. In total 33 protein sequences (Table 1) were investigated in phylogenetic as well as average distance analyses. Phylogenetic analysis allowed the classification of channels according to their evolutionary relationship, whereas the average distance analysis was performed on full-length sequences and sequence fragments to classify voltage-gated K^+^ channels with respect to certain criteria, like their rectification, for instance. The latter analyses allowed the exclusion of structural channel parts that are not responsible for the variability in channel gating.

**Table 1:** Overview of voltage-gated K^+^ channel sequences used in this study.

Channel name	Group		Species	Gene identifier
**Plant Kv-like channels**				
*Ath*-KAT1	plant	K_in_	*Arabidopsis thaliana*	AT5G46240
*Ath*-KAT2				AT4G18290
*Ath*-AKT1				AT2G26650
*Ath*-AKT5				AT4G32500
*Ath*-AKT6				AT2G25600
*Ath*-GORK	plant	K_out_		AT5G37500
*Ath*-SKOR				AT3G02850
*Ath*-AKT2	plant	K_weak_		AT4G22200
*Ath*-KC1	plant	regulatory subunit		AT4G32650
**HCN channels**				
*Hsa*-HCN1	animal	K_in_	*Homo sapiens*	O60741
*Hsa*-HCN2				Q9UL51
*Hsa*-HCN3				Q9P1Z3
*Hsa*-HCN4				Q9Y3Q4
*Mmu*-HCN1			*Mus musculus*	O88704
*Mmu*-HCN2				O88703
*Mmu*-HCN3				O88705
*Mmu*-HCN4				O70507
*Rno*-HCN1			*Rattus norvegicus*	Q9JKB0
*Rno*-HCN2				Q9JKA9
*Rno*-HCN3				Q9JKA8
*Rno*-HCN4				Q9JKA7
*Spu*-HCN			*Strongylocentrotus purpuratus*	NP_999729
**Kv channels**				
*Dme*-Shaker	animal	K_out_	*Drosophila melanogaster*	CAA29917
*Hsa*-Kv1.2			*Homo sapiens*	AAA36141
*Hsa*-Kv2.1				AAA36156
*Hsa*-Kv4.1				CAA06755
*Hsa*-Kv5.1				AAC05597
*Hsa*-Kv6.1				AAC05635
*Hsa*-Kv9.1				AAC13165
*Mmu*-Kv3.1			*Mus musculus*	CAA68814
*Rno*-Kv1.2			*Rattus norvegicus*	P63142
*Rno*-Kv2.1				P15387
*Rno*-Kv8.1				CAA67174

Furthermore, the inclusion of available crystal structures and homology protein models in the comparison enabled the investigation of channel attributes that are not accessible by the primary protein sequence, like distances between structural parts or intermolecular interactions.

### K^+^ channels of different function and origin vary most in the loop between S5 and the pore domain

**Fig 2:**
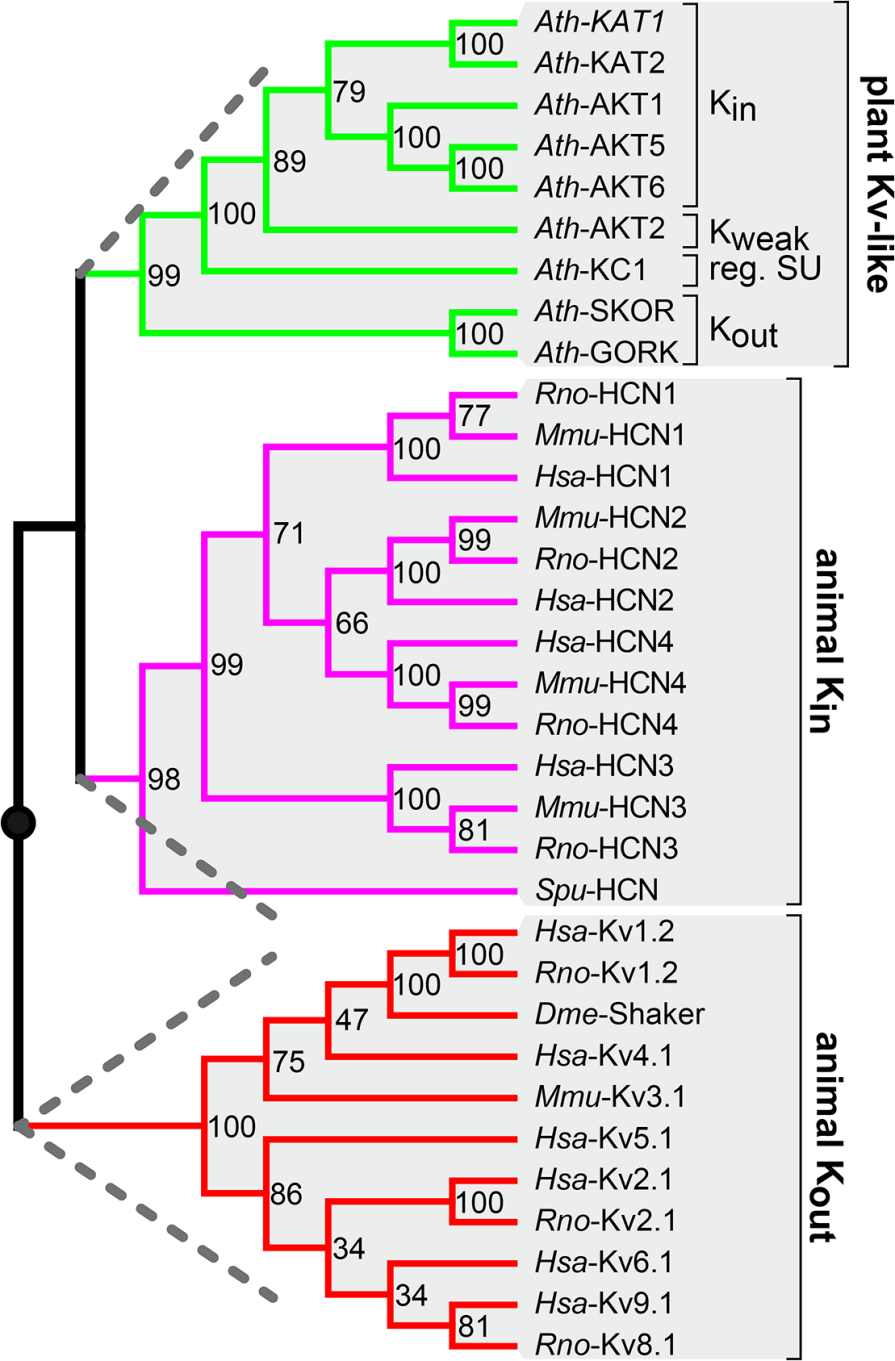
Plant Kv-like channels are closer related to animal K_in_ than to animal K_out_ channels. Phylogenetic analysis classifies voltage-gated K^+^ channels according to their function and appearance. The maximum likelihood tree was calculated using the WAG model and a bootstrap value of 1000. Plant Kv-like channels, animal K_in_ and animal K_out_ channels are each classified into one clade. Thereby, plant Kv-like and animal K_in_ channels belong to the same superior clade, while animal K_out_ channels are organized into a second superior clade. Dashed lines indicate the two mayor clades. Please note: The mid-point rooted tree allows conclusions about channel relationships, but not about assumptions of the ancestors.

The phylogenetic analysis grouped K^+^ channels into three major clades that coincide with the separation according to their origin and function (Fig 2). Plant voltage-gated K^+^ (plant Kv-like) channels form one clade, while the other two comprise animal K_in_ (HCN) and animal K_out_ (Kv) channels, respectively. Interestingly, plant Kv-like and animal K_in_ channels are sister clades indicating a close evolutionary relationship of both channel groups.

The multiple sequence alignment (MSA) shows similar sequence features that support the close relationship of HCN and plant Kv-like channels. The accuracy of the MSA was tested by annotating the position of transmembrane domains (TMD). The positions of TMD S1 to S6 identified according to their presence in available crystal structures and protein models were well aligned in the MSA (S1 Fig).

**Fig 3:**
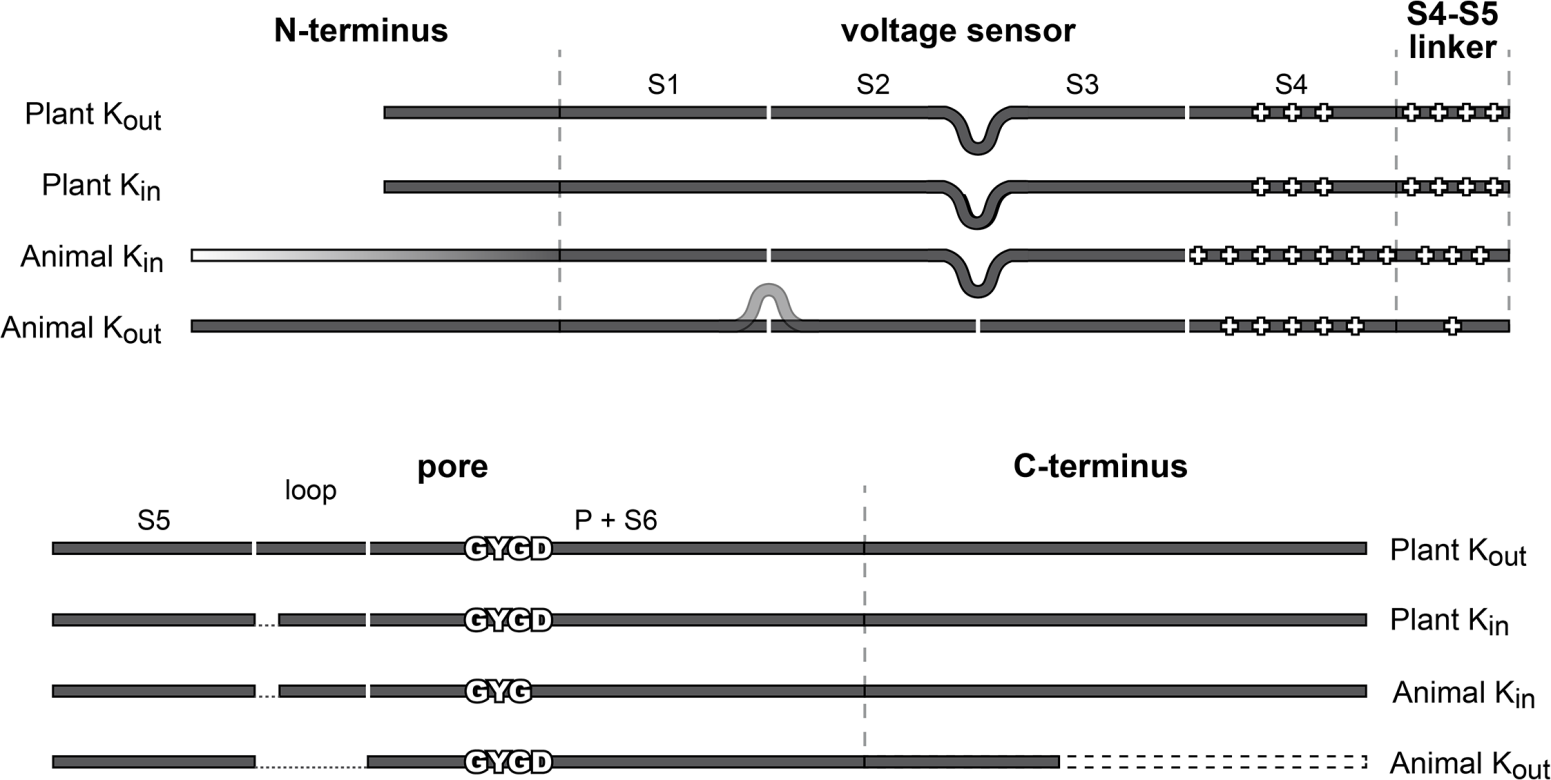
Simplified representation of structural characteristics of voltage-gated K^+^ channel groups. Voltage-gated K^+^ channel groups are defined according to the clades that arose from the phylogenetic analysis presented in Fig 2. The N-termini of these K^+^ channels are sparsely conserved. Even within channel groups there is a high variability in length (represented by the white to black gradient). Some animal K_out_ channels show an extended sequence between the first two TMDs. In contrast, all plant Kv-like and animal K_in_ channels have an extended loop between TMD S2 and S3. Animal K_in_ channels have the highest number of charged residues in S4, whereas the lowest number is present in plant Kv-like channels. In contrast, plant Kv-like channels feature the highest number of charged residues in the S4-S5 linker, while animal K_out_ channels hardly possess charged residues in this linker. The most striking structural difference that correlates with channel function is a loop between TMD S5 and the pore region. This loop is only present in plant Kv-like and animal K_in_ channels. In addition, a small stretch in the loop discriminates between plant K_in_ and K_out_ channels.

Structural features of defined K^+^ channel groups are illustrated in Fig 3. In general, the different channel classes are very distinct in their cytosolic parts, while the core transmembrane structures are rather similar. In the MSA the average conservation of positions within the protein core structures is more than 30 times higher than the average conservation of positions in the cytosolic channel parts (for details see Materials and Methods). Plant Kv-like and animal K_in_ channels had the longest C-termini with lengths of 295 to 680 amino acids, while among the considered animal K_out_ channels, only Kv2.1 contains a long C-terminus with comparable length (440 amino acids). All other animal K_out_ channels exhibit shorter C-termini ranging from less than 100 to 230 amino acids. In contrast, the N-termini are little conserved and variable even within the K^+^ channel groups.

In the more similar transmembrane core region, plant K_in_ and K_out_ as well as animal K_in_ channels have an extended loop of equal length between TMD S2 and S3 when compared to the other channels. The most striking difference, however, was the presence and length of a loop between TMD S5 and the pore domain. The S5-P loop is the only conserved structural difference that clearly distinguishes between animal K_in_ and K_out_ channels as well as between plant K_in_ and K_out_ channels, albeit not in a uniform manner. An extended S5-P loop is only present in animal K_in_ and plant Kv-like channels, but not in animal K_out_ channels. With about 16 amino acids, the S5-P loop has the same length in animal and plant K_in_ channels. In plant K_out_ channels, however, the loop is even longer with about 24 amino acids.

Further differences between main K^+^ channel groups were identified within the distribution of charged residues in TMD S4 and the S4-S5 linker. Plant Kv-like channels show the lowest number of charged residues in S4 (5–6), but the highest number in the S4-S5 linker (5–6). In contrast, animal K_out_ channels exhibit a higher number of charged residues in S4 (7–8), but hardly contain charged residues in the S4-S5 linker (1–2). When both structural parts are considered as a unit, animal K_in_ channels show the highest number of charged residues (~13). The variable number and distribution of charged residues in S4 and the linker may account for differences in the sensitivity towards the membrane voltage, but cannot explain the fundamental differences in coupling voltage-sensing to pore-opening; i.e. the opposite rectification behaviour.

### K^+^ channel rectification properties cannot be pinpointed to certain isolated channel parts

**Fig 4:**
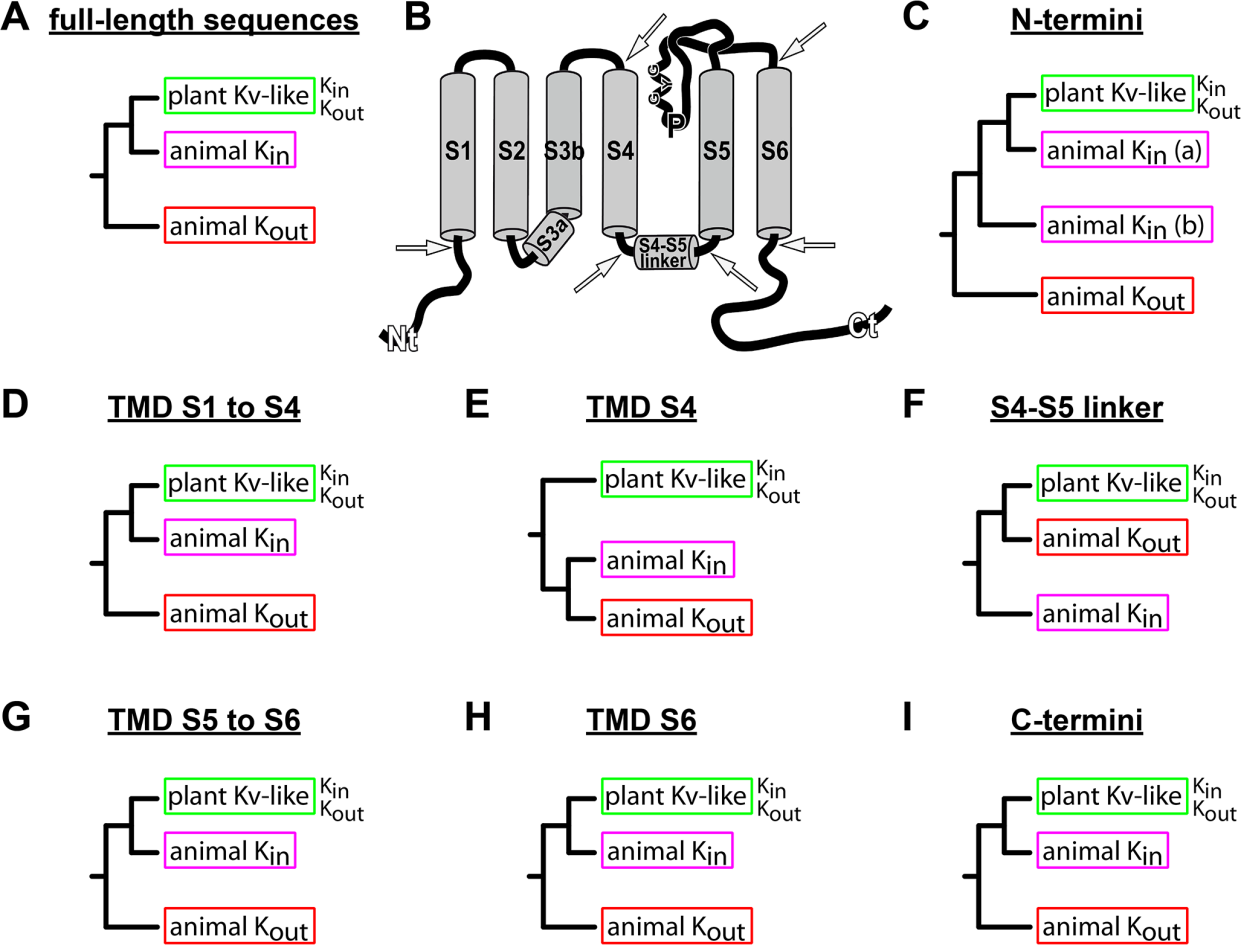
Simplified mid-point rooted trees based on average distance analyses of fragmented voltage-gated K^+^ channel sequences. (A) The simplified tree that represents the average distance analysis of full-length K^+^ channel sequences corresponds to the tree in Fig 2. (B) Arrows in the schematic channel representation mark separation points used for sequence fragmentation. Plant Kv-like and animal K_in_ channels cluster in one clade if only those channel structures are considered that code for S1 to S4 (D), S5 to S6 (G), only S6 (H), and the C-terminus (I). If only the N-termini are considered plant Kv-like channels cluster together with only some of the animal K_in_ channels (C). In contrast to the complete voltage sensor unit S1 to S4 (D), S4 alone does not differentiate between animal K_in_ and K_out_ channels (E). Here, animal K_in_ and K_out_ channels cluster together and plant Kv-like channels are present in a separated clade. The S4-S5 linker sequences are the only structural part that clusters plant Kv-like and animal K_out_ channels in one clade, which is separated from animal K_in_ channels (F).

K^+^ channel sequence clustering by phylogenetic analyses reflected well a separation according to their origin and their function (Fig 2). Fig 4A illustrates the result of Fig 2 as a simplified tree. To better illustrate the classification, only the organization in K^+^ channel groups is shown instead of displaying every K^+^ channel separately. In the following step we calculated average distances to allow channel clustering; here, instead of using full-length sequences, only channel fragments were compared: (i) only the N-termini, (ii) the TMDs S1 to S4, (iii) the isolated S4 segment, (iv) the S4-S5 linker, (v) the pore module comprising the TMDs S5 to S6, (vi) the isolated TMD S6, and (vii) the C-termini (Fig 4B; fragmentation points are indicated by white arrows). Average distance analysis performed on full-length sequences resulted in the same organisation of the K^+^ channels as seen for the phylogenetic analysis (S2 Fig).

On the basis of the regions S1-S4, S5-S6, S6 and the C-termini multiple sequence alignments followed by average distance analyses clustered K^+^ channels in a similar manner as observed for full-length sequences (Fig 4D, 4G, 4H and 4I). In all these cases, plant K_in_ and K_out_ channels were associated with animal K_in_ channels in the same clade. A slightly different picture arose when considering the isolated S4 segment, the S4-S5 linker or the N-terminus (Fig 4C, 4E and 4F). On the basis of S4 sequences, animal K_in_ and K_out_ channels clustered in a clade separated from plant K^+^ channels (Fig 4E). When considering only the S4-S5 linker, plant Kv-like and animal K_out_ channels were grouped together (Fig 4F). The classification based on N-terminal sequences was less strict, what is expected when taking into account the high variability in N-termini length (Fig 4C).

**Fig 5:**
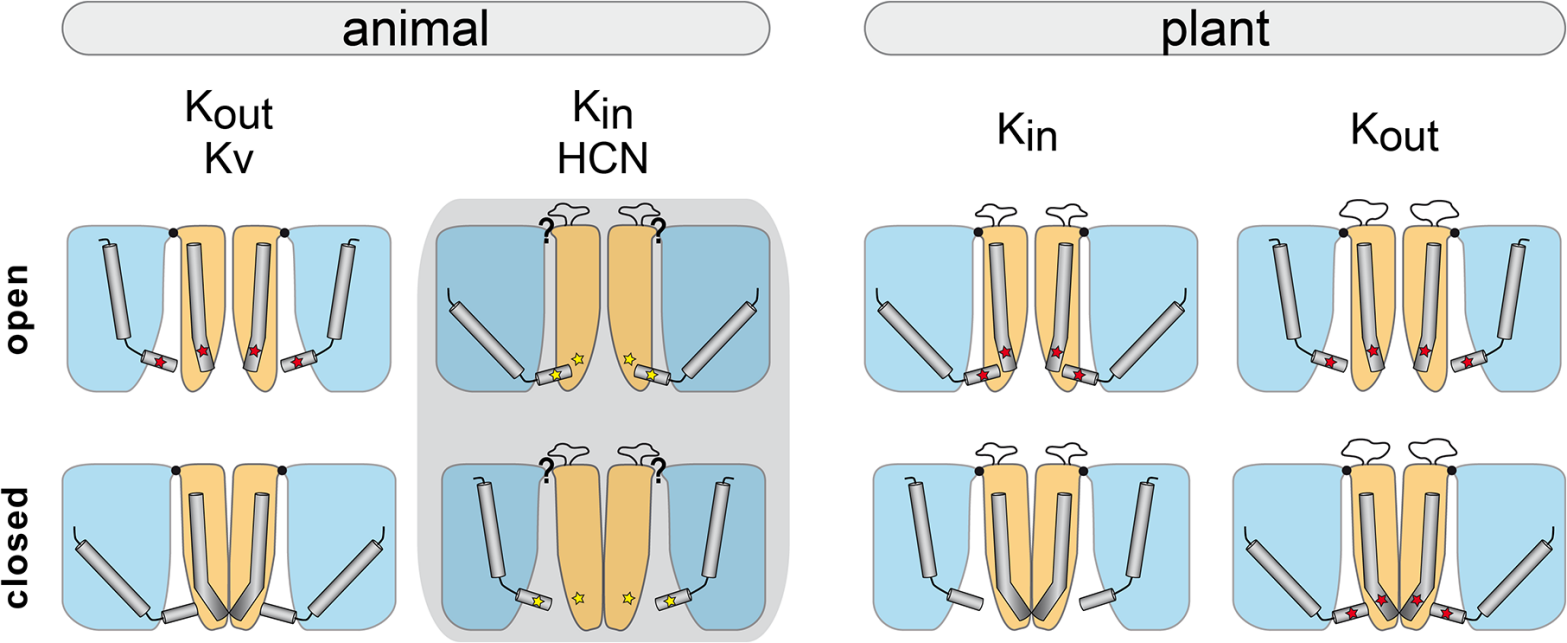
Illustration of voltage-gated K^+^ channel features described in Table 2. Channel characteristics are illustrated for animal and plant Kv-like channels in the open and in the closed state. The representation of animal K_in_ channels is grey shaded because no protein structures are available so far. In general, the voltage sensor is displayed in blue and the pore in orange. The pore opening at the intracellular side is according to the channel states wide or narrow. The width of this pore part correlates with the distance of the C-terminal S6 ends. S4 is marked within the voltage sensor in its up or down position, respectively. The S4-S5 linkers of all four subunits surround the inner part of the channel pore and form a kind of ring around it. Its diameter correlates with the S4 position and is larger when S4 is in the up position. A black dot at the extracellular side of Kv channels represents the occurrence of the connection of voltage sensor and pore. The loop between the C-terminal end of S5 and the pore TMD is displayed at the extracellular side of the pore in plant Kv-like and animal K_in_ channels. The bending of S6 is illustrated within the pore. It is more bended in the closed states and straighter in the open states. Red stars in plant Kv-like and animal K_out_ channels represent the interaction of the S4-S5 linker and S6. An interaction of these both structures has been described for animal K_in_ channels as well. But it is not clear in which state it appears. Therefore, a yellow star is used to illustrate the interaction.

The cluster analyses of sequence fragments of K^+^ channels allowed three main conclusions: (i) Plant K_in_ and K_out_ channels clustered always together, while animal K_in_ and K_out_ channels showed independent classification patterns. (ii) The charged TMD S4 alone did not contain sequence information that discriminated between different voltage-dependencies of channels, whereas the whole voltage sensor, S1-S4, did allow to distinguish between animal K_out_ channels on one side and animal K_in_ and plant Kv-like channels on the other. This observation confirmed experimental data proving that the voltage-dependent S4 movement in plant K_in_, animal K_in_ and animal K_out_ channels is similar and does not explain the opposite rectification properties (Figs 4 and 5 and Table 2, [19–21,29]. (iii) The S4-S5 linker did not comprise sequence information that discriminates between channel functions. The linker is a short stretch of a few amino acids that is tightly connected to S4. Therefore, the linker has to follow the universal S4 movement, and as a stand-alone structural element it could not explain the gating variability. Nevertheless, the S4-S5 linker is a structure with fundamental function in the gating process. Its peculiar importance derives from its interaction with the C-terminal part of S6 [8,10,30,31].

**Table 2:** Overview of characteristic features found in voltage-gated K^+^ channels of different taxa.

Channel / Group / Organism	Structure	State	S1-Pore connection	S4 position	S4-S5 linker diameter	S5-Pore loop	S6 bend (in °)	S6 ends distance (in Å)	S4-S5 linker and S6
**KAT1 /** plant K_in_ **/** *A*. *thaliana*	M	open	Yes^**+**^	down^E**+**^	smaller^**+**^	short^S^	155.9±1.5^**+**^	31.3±0.4^**+**^	connection^**+**^
	M	closed	yes^**+**^	up^E**+**^	larger^**+**^	short^S^	146.4±0.2^**+**^	22.5±0.3^**+**^	no connection^**+**^
**SKOR /** plant K_out_ **/** *A*. *thaliana*	M	open	yes^**+**^	up^**+**^	larger^**+**^	long^S^	154.1±0.6^**+**^	27.6±0.6^**+**^	connection^**+**^
	M	closed	yes^**+**^	down^**+**^	smaller^**+**^	long^S^	149.6±1.9^**+**^	22.7±0.6^**+**^	connection^**+**^
**HCN /** animal K_in_ **/** Mammalia + Echinoidea	**–**	open	n.a.	down^E^	n.a.	like plant K_in_^S^	n.a.	n.a.	interaction^E’^
	**–**	closed	n.a.	up^E^	n.a.	like plant K_in_^S^	n.a.	n.a.	interaction^E’^
**Kv1.2 /** animal K_out_ **/** *R*. *norvegicus*	C (2A79)/M	open	yes*	up^E^ *	larger^**+**^	**–** ^S^ *	150.9±0.0*	29.7±0.0*	connection^**+**^
	M	closed	yes^**+**^	down^E**+**^	smaller^**+**^	**–** ^S^ *	143.3±0.0^**+**^	24.1±0.0^**+**^	no connection^**+**^

Data derived from: + protein models

* crystal structures; E experiments; or S sequence analysis. ‘n.a.’ not available data due to lacking structure. E’ an interaction between the S4-S5 linker and the S6 end has been observed experimentally, but it is not clear in which state the interaction occurs.

M and C in the column ‘structure’ indicate availability of protein models (M) or crystal structures (C). Number given in brackets following the ‘C’ indicates PDB ID of the respective structure.

By all means, neither S4 nor the S4-S5 linker could explain the rectification behaviour of voltage-gated K^+^ channels. Thus, rectification properties seem to derive from structures surrounding S4 and the S4-S5 linker, rather than from these structures itself. Previous experimental work supports that information on the rectification behaviour is likely spread over these structures. There, we obtained experimental results supporting the interplay of various channel parts in the rectification process of plant Kv-like channels [28,32,33]. Domain swapping between plant K_in_ and K_out_ channels results in destruction of channel rectification or functionality, or does not affect the rectification at all. However, not a single K_in_-K_out_ chimera with inverted rectification properties has been identified so far. This led to the conclusion that the interplay of various channel parts is essential to obtain rectification.

### The shape of the sixth TMD correlates with conformational channel states

Next, we considered crystal structures of voltage-gated K^+^ channels and available homology models built on the basis of these structures [27,28,34]. In all these representations, the TMD S6 shows two structural characteristics that change during transition from the open to the close conformation and vice versa. The C-terminal ends of S6 bend around a glycine residue in plant Kv-like and animal K_in_ channels or a PxP motif in animal K_out_ channels [35–38]. Due to this bend, the ends of S6 can change their position and approach or depart from each other during channel state transition. In all K^+^ channel groups, the C-terminal S6 ends behaved in a conserved manner, they were closer in the closed conformation and more distant in the open conformation (Table 2 and Fig 5). The angle of the S6 bend also correlated with channel states. It was larger in the open conformation, which indicates that S6 is straightened during channel opening.

### Two ubiquitous interfaces connect voltage sensor and pore but exhibit channel-class specific differences

In general, the voltage-sensing module and the pore module are structurally connected via a short amino acid sequence at the intracellular side of the channel–the S4-S5 linker. A second interface has been described at the extracellular side between the C-terminal end of S1 and the junction between TMD S5 and the pore domain [11]. This connection is formed by interacting amino acid side chains. The examination of structures of channel proteins in the open and closed conformation showed that in all K^+^ channel groups certain amino acids in the C-terminal region of S1 were in close proximity (<3Å) to the S5-P region (Table 2 and S3 Fig).

Although these two links appeared to be ubiquitous features of voltage-gated K^+^ channels, there were channel class-specific differences: (i) The extracellular S1-S5/P connection involved regions that were shown to discriminate between K^+^ channel groups (Figs 3 and 5). It is thus likely that the functional features of this junction can be distinct in the different groups. However, with the available data it is not clear whether the S5-P loop is directly involved in this interaction or whether this interaction restricts rather to close-by sequence regions. For animal K_out_ channels this connection has been identified by analysing Kv channel sequences and its importance has been experimentally confirmed by the examination of Shaker channel mutants [11]. The plant Kv-like channel models do not contain the S5-P loop, because they have been modelled on the bases of the animal K_out_ channel that lacks this loop. However, domain swaps of the loop between the plant Kv-like channels KAT1 and SKOR showed its critical role for channel functionality [32,33,39]. (ii) At the intracellular side, the S4-S5 linker interacts with the C-terminal end of S6 as shown experimentally and on the basis of structural data (Table 2) [5,8,9,33,40]. Molecular modelling data suggested that this interaction was not the same in all voltage-gated K^+^ channels. Protein models of plant Kv-like channels proposed a permanent connection between S4/S5 and S6 in the case of the K_out_ channel SKOR. In contrast, in the case of the K_in_ channel KAT1 the connection was found in the open state only. Interestingly, also the crystal structure of the animal K_out_ channel chimera Kv1.2/2.1 shows the S4/S5-S6 connection only in the open but not in the closed channel state [40]. In this context animal K_out_ channels appear to be like plant K_in_ channels.

For animal K_in_ channels on the other hand it has been shown that the so called C-linker right after S6 is interacting with residues from the S4-S5 linker and has significant impact on the correct coupling between voltage-sensor and pore module [41,42]. The C-linker of animal K_in_ channels consists of six α-helices that connect S6 with the C-terminus. The helix closest to S6, the A’ helix, is located underneath the channel and is able to interact with the S4-S5 linker. In animal K_out_ channels the C-termini are rather short. Instead, the N-termini are pronounced and interact underneath the channel [34]. In the case of plant Kv-like channels C-terminal structural data is missing. However, experimental data provide room for speculation that plant K_in_ and K_weak_ channels might share the same voltage-sensor and pore coupling like animal K_in_ channels (reviewed in [43]).

Taken together, the behaviour of the voltage-sensing domain S4 and the pore-lining domain S6 were conserved among inward- and outward-rectifying K^+^ channels in animals and plants. The two connections of voltage sensor and pore were conserved as well, but bear channel-class specific differences. These differences might provide variability in the flexibility of the overall K^+^ channel structure, which in turn results in an unequal forwarding of the universal voltage sensor movement to the pore.

### Plant K_in_ and K_out_ channels are both more closely related to animal K_in_ channels than to animal K_out_ channels

Structural and cluster analyses clearly pointed to differences that allow discriminating between animal K_in_ and K_out_ channels. Such segregation into the two functional types was not possible for plant Kv-like channels. Phylogenetic and average distance analyses always grouped all plant Kv-like channels within the same clade, despite their functional differences (Figs 2 and 4). For the plant K_weak_ channel AKT2 this observation is not surprising. As it has been described as specialised K_in_ channel that switches between K_in_ and K_weak_ channel properties dependent on its phosphorylation status [44–46]. Plant K_out_ channels on the other hand exhibit a contrary physiological function. Surprisingly, they were included in the K_in_ channel clade rather than in the K_out_ channel clade indicating that plant K_out_ channels are structurally closer to K_in_ channels. This implies that plant and animal K_out_ channels bear structural differences that do not affect the physiological function of K^+^ efflux. To solve this paradoxical evolutionary and functionally classification we propose that animal K_out_ and plant K_out_ channels occurred independently of each other in life history. In other words this would mean that the feature of outward-rectification of K^+^ channels has been invented twice in evolution.

**Fig 6:**
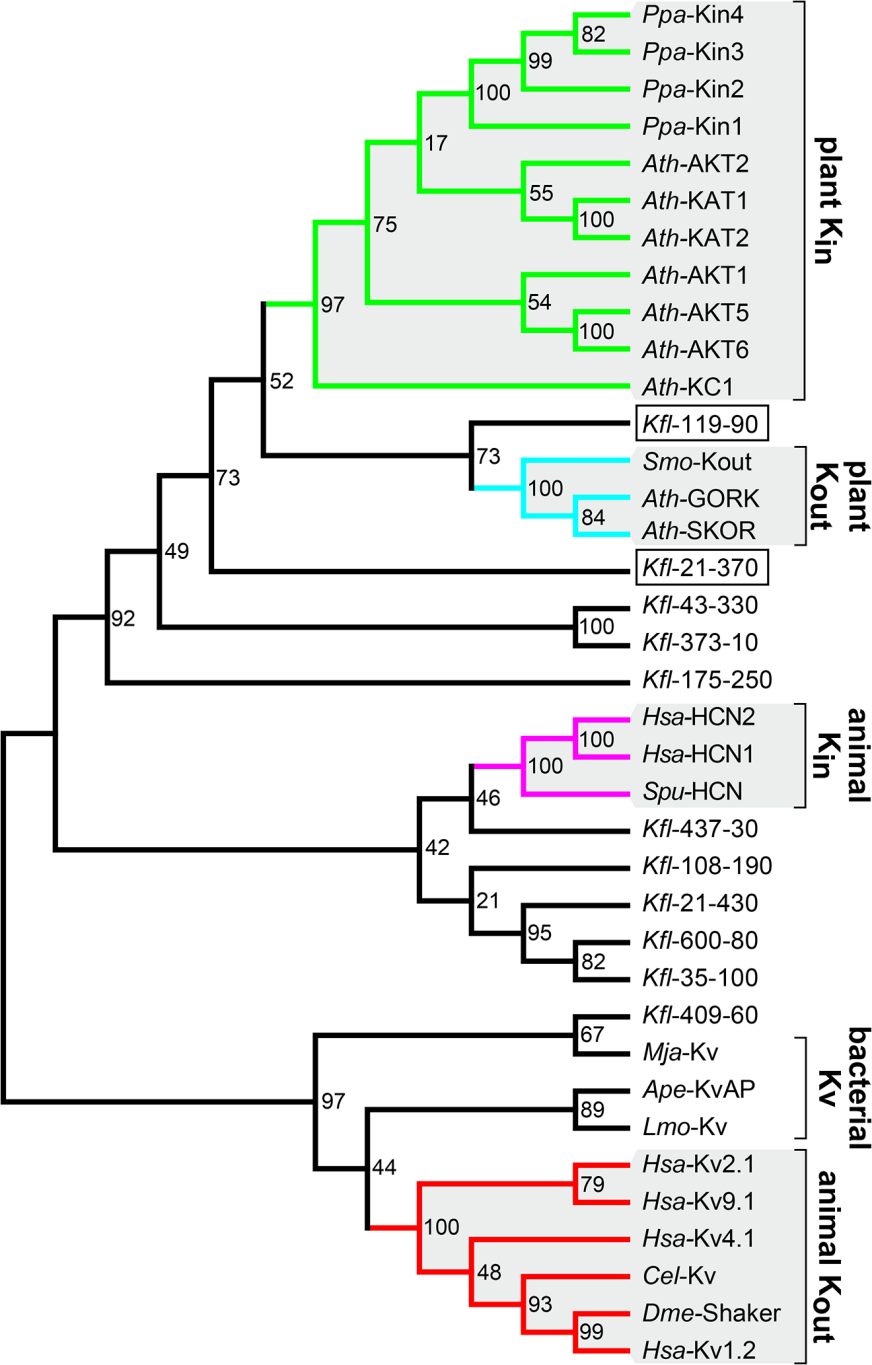
Two *Klebsormidium* voltage-gated K^+^ channels are closely related to Kv-like channels of higher land plants. A maximum likelihood tree was calculated using the WAG model and a bootstrap value of 1000 with K^+^ channel sequences from (i) bacteria (*Aeropyrum pernix*: *Ape*-KvAP (APE_0955); *Listeria monocytogenes*: *Lmo*-Kv (lmo2059); *Methanocaldococcus jannaschii*: *Mja*-Kv (MJ0139)), (ii) animals (see Table 1; *Caenorhabditis elegans*: *Cel*-Kv (CELEg_ZK1321.2)), (iii) early land plants (*Selaginella* moellendorffii: *Smo*-Kout (scaffold_121_2_ORF2559)); *Physcomitrella patens*: *Ppa*-Kin1 (Pp1s3_156V6.1), *Ppa*-Kin2 (Pp1s2_170V6.1), *Ppa*-Kin3 (Pp1s22_165V6.1), *Ppa*-Kin4 (Pp1s283_74V6.1), (iv) eudicots (see Table 1), and (vi) the early terrestrial alga *Klebsormidium* (*Kfl*-21-370 (kfl00021_0370), *Kfl*-21-430 (kfl00021_0430), *Kfl*-35-100 (kfl00035_0100), *Kfl*-43-330 (kfl00043_0330), *Kfl*-108-190 (kfl00108_0190), *Kfl*-119-90 (kfl00119_0090), *Kfl*-175-250 (kfl00175_0250), *Kfl*-373-10 (kfl00373_0010), *Kfl*-409-60 (kfl00409_0060), *Kfl*-437-30 (kfl00437_0030), *Kfl*-600-80 (kfl00600_0080)). The phylogenetic tree is mid-point rooted and displays the relationship of K^+^ channels. The main K^+^ channel families are grouped in separated clades. Plant K_in_ and K_out_ channels as well as animal K_in_ and K_out_ channels form individual clades. Thereby, bacterial and animal K_out_ channels are separated from K^+^ channels from green plants as well as animal K_in_ channels. The K^+^ channels from *Klebsormidium* are not grouped into one clade. Instead they spread over the entire tree indicating a broad diversity of different K^+^ channels. Especially two, out of eleven, K^+^ channels from *Klebsormidium* (marked by a square) are closely related to plant K_in_ and plant K_out_ channels.

**Fig 7:**
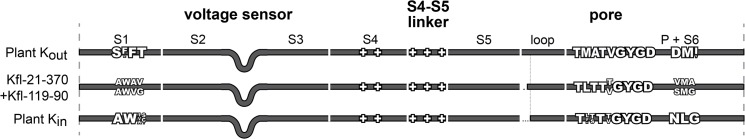
Simplified representation of structural features of Kv-like channels from higher land plants and *Klebsormidium*. The core channel parts S1 to S6 are displayed including features that are characteristic for these K^+^ channel groups, like the extended S2-S3 and S5-P loops, conserved positive charges in S4 and the S4-S5 linker, and the selectivity filter motif TxxTxGYGD. Furthermore, two motifs in the transmembrane domains S1 and S6 are displayed. Outward-rectifying K^+^ channels contain an SFFT motif in S1, where inward-rectifying channels contain an AWxx motif. In S6 the characteristic DMI (K_out_ channels) and NLG (K_in_ channels) motifs are illustrated. In Klebsormidium the S6 motif is not yet specified, while the motif in S1 is that of K_in_ channels. The extended loop between S5 and P is two amino acids longer than in plant K_in_ channels and six amino acids shorter than the corresponding loop in plant K_out_ channels.

To challenge this hypothesis in an extended phylogenetic analysis, we included sequences of voltage-gated K^+^ channels from (i) the moss *Physcomitrella patens*, (ii) the ancient vascular plant species *Selaginella moellendorffii*, (iii) the freshwater alga *Klebsormidium flaccidum*, (iv) bacteria and (v) *Caenorhabditis elegans* (Fig 6). The phylogenetic analysis corroborated the close relationship of plant Kv-like channels to animal K_in_ channels, while bacterial and animal K_out_ channels form a distant group. Interestingly, the eleven K^+^ channels found in the oldest of the considered plants, *K*. *flaccidum*, are not clustered in one clade. Instead they spread over the entire tree. Two of them, *Kfl*-21-370 and *Kfl*-119-90, are closely related to plant Kv-like channels sharing ~40% identity in the channel core region. Indeed, *Kfl*-21-370 and *Kfl*-119-90 show several features that are exclusive characteristics of plant Kv-like channels, like a comparable number of charged residues in S4 and the S4-S5 linker, additional amino acids between S2 and S3 as well as the longer loop between S5 and the pore domain (Fig 7). Interestingly, from a structural point of view, the *Klebsormidium* K^+^ channels are intermediates between plant K_in_ and K_out_ channels: (i) In its core region *Kfl*-21-370 is 41% identical to the plant K_in_ prototype KAT1 and 35% to the K_out_ channel SKOR. Likewise, *Kfl*-119-90 is 43% identical to KAT1 and 42% to SKOR. (ii) The length of the S5-P loop is two amino acids longer than in plant K_in_ channels and six amino acids shorter than in plant K_out_ channels. (iii) A characteristic motif in TMD S6 (DMI in K_out_ channels; NLG in K_in_ channels), which is important for the K^+^ sensing mechanism of plant K_out_ channels [39], is not yet developed in *Klebsormidium* K^+^ channels. (iv) In TMD S1 *Kfl*-21-370 and *Kfl*-119-90 share a motif with plant K_in_ channels. (v) The expanded K^+^-selectivity filter (TxxTxGYGD) is that of plant K_in_ channels and at the x-positions clearly different from the highly conserved motif of plant K_out_ channels. Taken together, the two K^+^ channels from *Klebsormidium* exhibit more plant specific structural channel features than Kv-like channels from bacteria or animals, and they are slightly closer to plant K_in_ channels than to plant K_out_ channels. Thus, it is likely that plant K_in_ and K_out_ channels evolved in the plant lineage from a common ancestor. This assumption is further encouraged by the studies performed by Gomez-Porras and colleagues [47]. They demonstrated in comprehensive phylogenetic analyses that the transition of green plants from an aqueous to a terrestrial environment was attended by a dramatic reduction of the K^+^ channel diversity. The fact that structural K_in_ features can be found in K^+^ channels from mosses and algae but typical structural K_out_ features can first be found in K^+^ channels from vascular plants suggests that plant K_out_ channels evolved from K_in_ channels.

## Conclusion

The comparison of voltage-gated K^+^ channels of different kingdoms revealed new insights into the structural and evolutionary background of K^+^ channel rectification. The entire plant Kv-like channel family likely emerged from a common ancestor and is closer related to animal K_in_ than to animal K_out_ channels. Thus, plants probably developed outward-rectification of Kv-like channels anew and independently of the outward-rectification that is found in animals. Several structural differences and similarities supporting this finding have been identified throughout voltage-gated K^+^ channel groups. Two known voltage sensor and pore interfaces have been observed as ubiquitous traits in voltage-gated K^+^ channels, the S4-S5 linker on one hand and the connection of S1 with the S5-P region on the other hand; even though, both interfaces bear channel class-specific differences in their specifications. S1 forms constant interactions with or close to the variable S5-P loop, while the S4-S5 linker establishes variable interactions with the C-terminal end of S6. All in all, several smaller structural differences appear to play together to provide variable flexibilities to voltage-gated K^+^ channel structures, which may result in variable transitions of the voltage sensor movement to the pore and therefore in an inverse coupling of voltage sensor and pore opening in K_in_ and K_out_ channels.

## Supporting Information

S1 Fig Multiple sequence alignment created for phylogenetic and cluster analyses of plant Kv-like and animal HCN and Kv channels. Displayed are the core regions only (TMD S1-S6) with indication of TMD positions of KAT1 (plant K_in_), SKOR (plant K_out_) and Kv1.2 (rat). Amino acids are coloured according to the colouring method used by Clustal. The colour density correlates with the conservation of a residue in each column. The more intense a colour, the higher is the conservation of a given amino acid. For orientation the approximate positions of the TMDs S1 to S6 and the pore domain P are indicated.
(PDF)

S2 Fig Average distance analysis clusters voltage-gated K^+^ channels just as phylogenetic analysis does. Calculating the average distance using the BLOSUM62 matrix clustered K^+^ channels in three main groups. As seen in the phylogenetic analysis plant Kv-like channels, animal K_in_ and animal K_out_ channels cluster each in one clade. Thereby, plant Kv-like and animal K_in_ channels are sister clades.
(TIF)

S3 Fig Residues in close proximity of the extracellular VSD-pore interface. Residues of the C-terminal S1 and the S5-P region that are situated in a radius of 3Å or less have been calculated in molecular dynamics simulations of the plant K_in_ channel KAT1 and the plant K_out_ channel SKOR, as well as in the protein structure of Kv1.2. In analyses of the KAT1 and SKOR models those contacts are marked that appear in more than 80% of the trajectories (grey boxes).
(TIF)
