# Supplementary material for: Outward Rectification of Voltage-Gated K+ Channels Evolved at Least Twice in Life History
Source: PLoS One. 2015 Sep 10;10(9):e0137600. doi: 10.1371/journal.pone.0137600 (PMC4565715; doi:10.1371/journal.pone.0137600)
Supplement: S1 Fig — Displayed are the core regions only (TMD S1-S6) with indication of TMD positions of KAT1 (plant Kin), SKOR (plant Kout) and Kv1.2 (rat). Amino acids are coloured according to the colouring method used by Clustal. The colour density correlates with the conservation of a residue in each column. The more intense a colour, the higher is the conservation of a given amino acid. For orientation the approximate positions of the TMDs S1 to S6 and the pore domain P are indicated. (PDF) [file pone.0137600.s001.pdf]

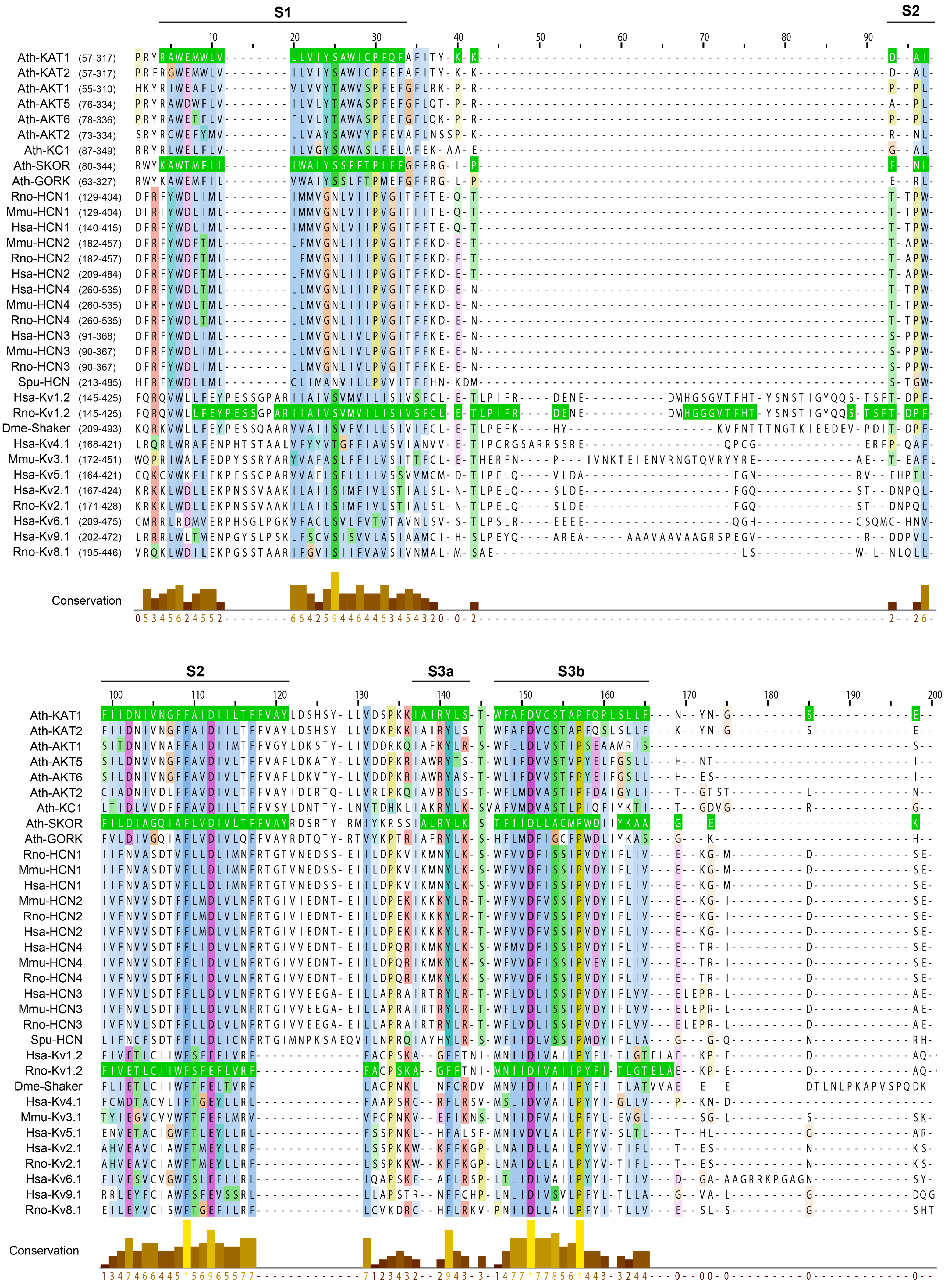
S1
S2
Ath-KAT1 (57-317)
Ath-KAT2 (57-317)
Ath-AKT1 (55-310)
Ath-AKT5 (76-334)
Ath-AKT6 (78-336)
Ath-AKT2 (73-334)
Ath-KC1 (87-349)
Ath-SKOR (80-344)
Ath-GORK (63-327)
Rno-HCN1 (129-404)
Mmu-HCN1 (129-404)
Hsa-HCN1 (140-415)
Mmu-HCN2 (182-457)
Rno-HCN2 (182-457)
Hsa-HCN2 (209-484)
Hsa-HCN4 (260-535)
Mmu-HCN4 (260-535)
Rno-HCN4 (260-535)
Hsa-HCN3 (91-368)
Mmu-HCN3 (90-367)
Rno-HCN3 (90-367)
Spu-HCN (213-485)
Hsa-Kv1.2 (145-425)
Rno-Kv1.2 (145-425)
Dme-Shaker (209-493)
Hsa-Kv4.1 (168-421)
Mmu-Kv3.1 (172-451)
Hsa-Kv5.1 (164-421)
Hsa-Kv2.1 (167-424)
Rno-Kv2.1 (171-428)
Hsa-Kv6.1 (209-475)
Hsa-Kv9.1 (202-472)
Rno-Kv8.1 (195-446)
Conservation
S2
S3a
S3b
Conservation

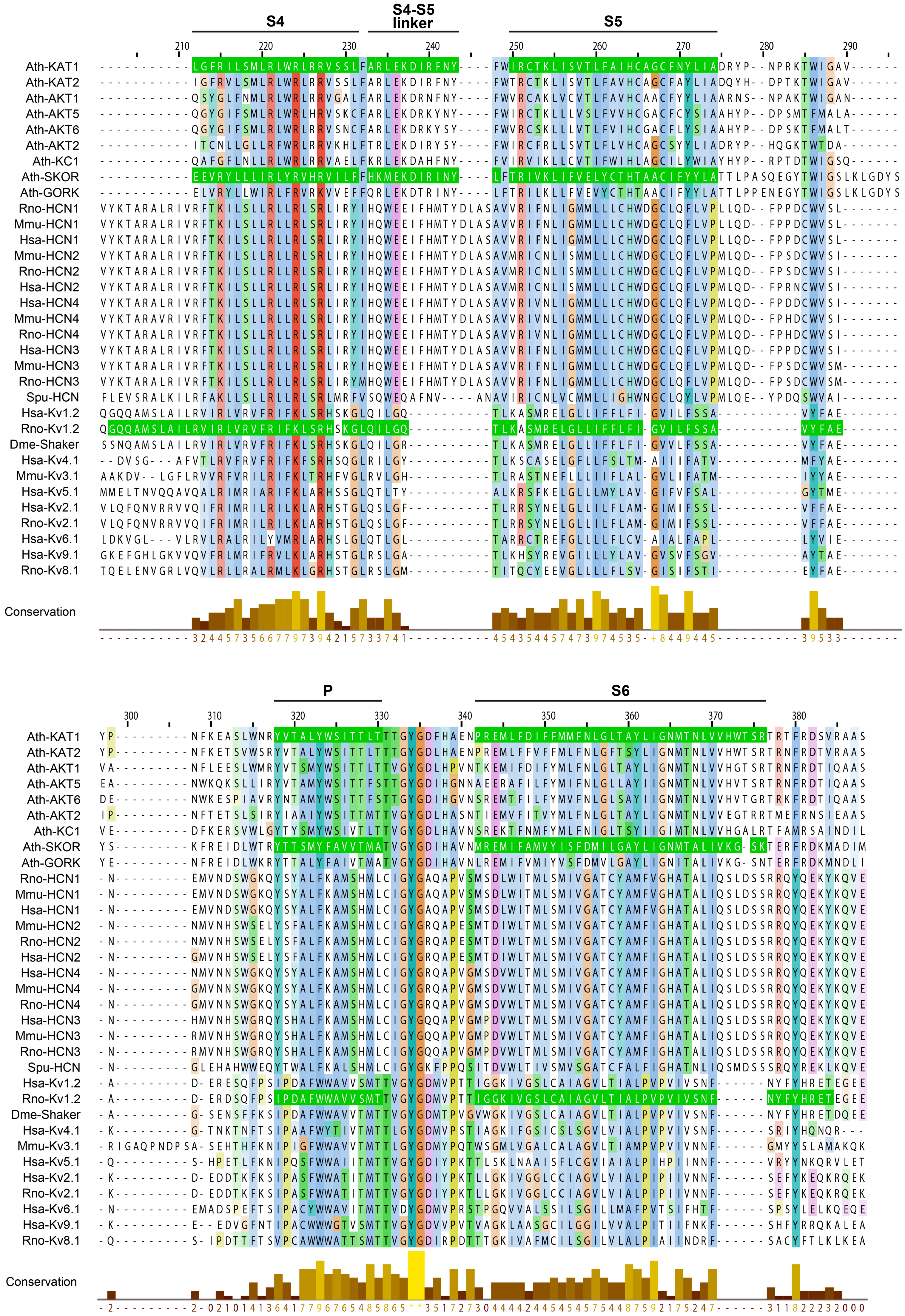
S4
S4-S5 linker
S5
210
220
230
240
250
260
270
280
290
Ath-KAT1
Ath-KAT2
Ath-AKT1
Ath-AKT5
Ath-AKT6
Ath-AKT2
Ath-KC1
Ath-SKOR
Ath-GORK
Rno-HCN1
Mmu-HCN1
Hsa-HCN1
Mmu-HCN2
Rno-HCN2
Hsa-HCN2
Hsa-HCN4
Mmu-HCN4
Rno-HCN4
Hsa-HCN3
Mmu-HCN3
Rno-HCN3
Spu-HCN
Hsa-Kv1.2
Rno-Kv1.2
Dme-Shaker
Hsa-Kv4.1
Mmu-Kv3.1
Hsa-Kv5.1
Hsa-Kv2.1
Rno-Kv2.1
Hsa-Kv6.1
Hsa-Kv9.1
Rno-Kv8.1
Conservation
P
S6
300
310
320
330
340
350
360
370
380
Conservation
