# Supplementary figures and images for: Outward Rectification of Voltage-Gated K+ Channels Evolved at Least Twice in Life History
Source: PLoS One. 2015 Sep 10;10(9):e0137600. doi: 10.1371/journal.pone.0137600 (PMC4565715; doi:10.1371/journal.pone.0137600)

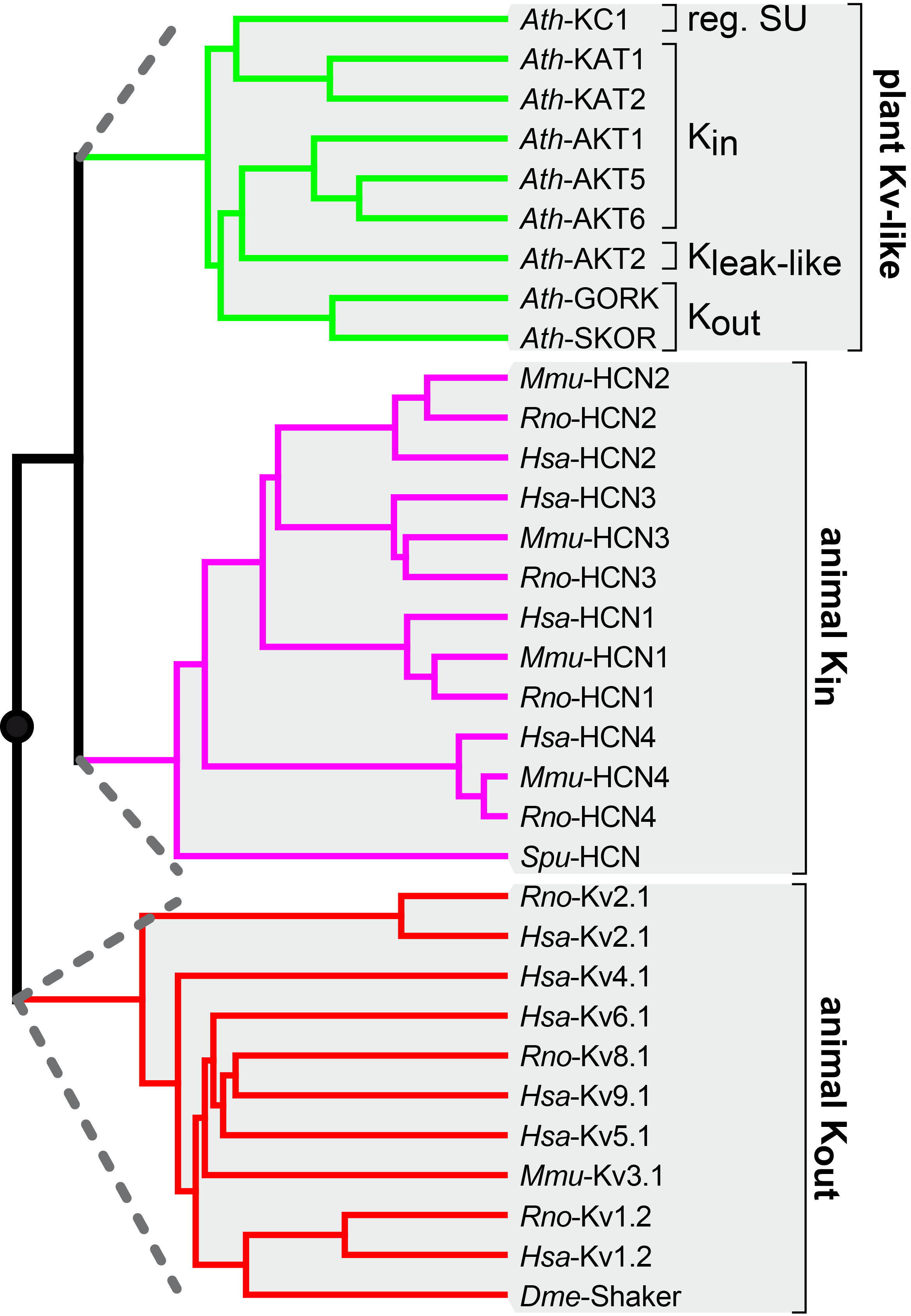

Supplement: S2 Fig — Calculating the average distance using the BLOSUM62 matrix clustered K+ channels in three main groups. As seen in the phylogenetic analysis plant Kv-like channels, animal Kin and animal Kout channels cluster each in one clade. Thereby, plant Kv-like and animal Kin channels are sister clades. (TIF) [file pone.0137600.s002.tif]

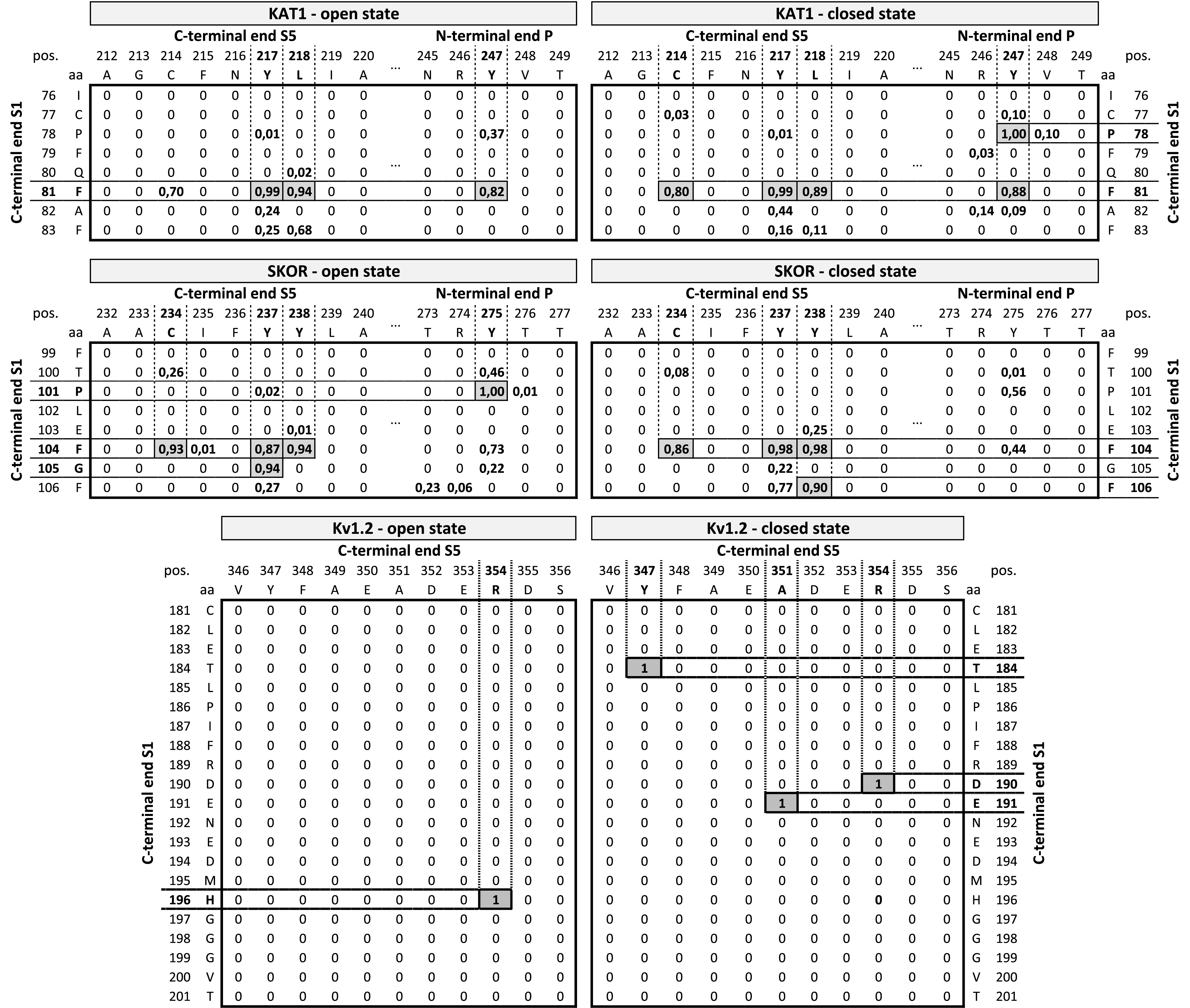

Supplement: S3 Fig — Residues of the C-terminal S1 and the S5-P region that are situated in a radius of 3Å or less have been calculated in molecular dynamics simulations of the plant Kin channel KAT1 and the plant Kout channel SKOR, as well as in the protein structure of Kv1.2. In analyses of the KAT1 and SKOR models those contacts are marked that appear in more than 80% of the trajectories (grey boxes). (TIF) [file pone.0137600.s003.tif]
